# Real-Time RISC-V-Based CAN-FD Bus Diagnosis Tool

**DOI:** 10.3390/mi14010196

**Published:** 2023-01-12

**Authors:** Cosmin-Andrei Popovici, Andrei Stan

**Affiliations:** Department of Computer Engineering, Faculty of Automatic Control and Computer Engineering, “Gheorghe Asachi” Technical University of Iași, 700050 Iași, Romania

**Keywords:** hardware acceleration, RISC-V, CAN-FD, SoC, FPGA, ethernet, UDP, communication

## Abstract

Network Diagnosis Tools with industrial-grade quality are not widely available for common users such as researchers and students. This kind of tool enables users to develop Distributed Embedded Systems using low-cost and reliable setups. In the context of RISC-V Extensions and Domain-Specific Architecture, this paper proposes a Real-Time RISC-V-based CAN-FD Bus Diagnosis Tool, named RiscDiag CanFd, as an open-source alternative. The RISC-V Core extension is a CAN-FD Communication Unit controlled by a dedicated ISA Extension. Besides the extended RISC-V core, the proposed SoC provides UDP Communication via Ethernet for connecting the proposed solution to a PC. Additionally, a GUI application was developed for accessing and using the hardware solution deployed in an FPGA. The proposed solution is evaluated by measuring the lost frame rate, the precision of captured frames timestamps and the latency of preparing data for Ethernet communication. Measurements revealed a 0% frame loss rate, a timestamp error under 0.001% and an acquisition cycle jitter under 10 ns.

## 1. Introduction

The fundamental problem that arose in the field of integrated circuits design and fabrication at beginning of this century is the fact that the classical laws (or the hypothesis today) of VLSI (Very Large-Scale Integration) became almost impossible to be applied anymore. The number of transistors in an integrated circuit cannot be doubled every two years from more than a decade nowadays (Moore’s Law [1]), and the power density will not remain the same for embedding more transistors in the same silicon area (Dennard’s Scaling [2]). These physical limitations of the classical performance improvement schema, which was applied for decades in the previous century, are not the only problems that the engineers are dealing with.

A lot of modern domains, which are planned to improve or already improve our life quality, such as IoT (Internet of Things), Autonomous Driving and ADAS (Advanced Driving Assistance Systems), Smart Home, Smart Cities, Electrical Vehicles and XaaS (Everything-as-a-Service), need High-Performance Computing and Ultra-High-Speed Communications to achieve the expected impact. Those domains are pushing the engineers and physicists to find new solutions for delivering an increased speed of computations for an unprecedented amount of data and extremely high-speed communication, all performed at a low power consumption.

A major step forward for the Computer Architecture domain was made in 2014 when Krste Asanovic and David Patterson from Berkeley University advanced the following question: “why is one of the most important interfaces proprietary?” [3], referring to ISAs, and proposed an open ISA (Instruction Set Architecture) and the associated architecture called RISC-V. The RISC-V project empowered many academic and industrial players to work together for accelerated development in the field of Computer Architecture and opened the door for new types of multicore processors with a general purpose [4] or application-specific extensions [5].

In a previous paper, the authors proposed and extended an architecture of the RISC-V Core which embeds a CAN-FD communication unit named RisCanFd CPU, which proved to be three times faster than an ARM Cortex-M7 microcontroller with a classical CAN-FD memory-mapped peripheral working at 6 times higher frequency. From our experience and research, a communication RISC-V extension for controlling a CAN-FD interface has not been developed yet, or, if they exist, nothing about them is public due to the industry proprietary nature of the projects. In this paper, the solution is extended for illustrating a real use case and not just the benefits of an RISC-V extension over classical CAN-FD memory-mapped peripherals [6].

The CAN-FD communication buses are present in most of the cars nowadays besides other high-speed protocols such as Automotive Ethernet and connect tens of ECUs placed in the modern vehicles for ensuring premium functionalities such as ADAS (Advanced Driving Assistance Systems), Autonomous Driving and Electrical Mobility. They extend the CAN 2.0 protocol by permitting the payload to be transmitted at higher bit rates of a maximum of 8 Mbps instead of 1 Mbps, and the maximum payload length became 64 instead of 8. Diagnosing such large CAN-FD communication buses, which are almost permanently used at a 100% busload, may become a challenging task for classical microcontrollers containing CAN-FD-capable peripherals. The technologies, devices and software licenses necessary for this purpose are sold at high prices, are affordable only for companies and are kept as closed IPs (Intellectual Property) by their producers such as Vector, Kvaser, Peak Systems, National Instruments and others.

In this paper, an open-source alternative for these proprietary solutions is presented. The real-time RISC-V-based CAN-FD bus diagnosis tool enriches the SoC (System-on-a-Chip) presented in [6] with the hardware logic needed for transferring all the CAN-FD frames exchanged by the ECUs (Electronic Control Units) on the bus to a PC, together with the information associated with bus error situations which may occur. The SoC is called RiscDiag CanFd and communicates with the PC via a dedicated Ethernet port using UDP (User Datagram Protocol) as the transport layer protocol for transferring captured frames and bus error information from the SoC to the user’s computer.

All the data acquired from the CAN-FD bus and sent to the PC is displayed to the user and placed within trace files by using a GUI (Graphical User Interface) application named RiscDiag_CanFd_App developed in C# under .NET Framework 4.8.

Besides this introduction representing the first chapter, in the second chapter, the work related to the current paper will be briefed. The third chapters will offer detailed information on the Hardware Architecture of the solution (the RiscDiag CanFd SoC) and will also cover the Software Architecture of the RiscDiag_CanFd_App running on the PC connected to the Hardware Solution via Ethernet. The fourth chapter will introduce the readers to the tests and measurements performed for evaluating and validating the solution.

## 2. Related Work

The quest for the computing systems’ performance improvement has recently been shifted towards Domain-Specific Architectures (DSAs), where the performance increase is due to the addition of custom computing units that are designed to perform very specific tasks in a very efficient manner. There are multiple domains that motivated the development of custom digital units that can accelerate the processing for specific applications: neural network processing, cryptographic computations and custom arithmetic processing.

Computing systems are involved in almost every aspect of human activities and have a direct or indirect impact on the performance and quality of the systems that rely on the provided results and computations. The themes of computing technology impact are exhaustively presented in [7] and include societal, technical and business dimensions. The need for continuous performance improvement drives the current research and development in computer engineering, and we are currently witnessing a design paradigm shift to DSAs, as presented in [8]. The classic design paradigm that fueled the innovations in the last 60 years was based on transistor improvements, microarchitectural improvements such as pipelines and superscalar execution and, lately, on multiprocessing. These methods lead to a spectacular performance increase in the general-purpose computing systems, but they reached a point where the power consumption does not allow for further improvement. The path to follow to obtain an additional and significant performance increase is developing DSAs that perform just a few tasks (i.e., are not general-purpose anymore) but do so extremely well [9]. One of the first industry-proven accelerators is the Tensor Processing Unit (TPU) [10] used by Google in its datacenters to speed up the inference phase of Deep Neural Networks by 10x. Additionally, a power dissipation decrease is observed compared to general-purpose CPUs and GPUs that perform similar tasks.

RISC-V is a standardized Instruction Set Architecture (ISA) specification [11] that explicitly provides the possibility to extend the base instruction set with custom extensions that use (i.e., abstract) custom-designed accelerators. There is a recent and significant academic body of work that uses the RISC-V specification as a tool to design custom accelerators to improve specific classes of algorithms and applications.

The design of algorithms for Neural Networks (NN) is one of the most targeted domains for building accelerators. In article [12], the authors propose an instruction set extension that accelerates the dot product operation and loop control in a constrained environment (i.e., embedded system) built with an RISC-V microprocessor. They report a 73% decrease in computing time (i.e., latency) and a power reduction by 73%. The authors of article [13] extend the base RISC-V instruction set with SIMD (Single Instruction-Multiple Data) instructions for low-bit-count operands and report a 6× and 8× speedup of QNN (Quantized Neural Networks) convolution kernels. Another extension is presented in article [10], which uses a new floating point number representation and an RISC-V extension that uses that representation and achieves speedups of up to 10× on inference time. In article [14], the authors design a custom AI system which includes multiple AI cores and evaluate it with an FPGA prototype using image and speech recognition testcases. They consider various NN topologies and vector sizes for accuracy comparison. The acceleration of neural networking workloads is presented in paper [15], where the authors design an extension of the base RISC-V ISA that allows for the conversion between 8- or 16-bit posits and 32-bit IEEE Floats or fixed point formats in order to offer a compressed representation of real numbers with little-to-no accuracy degradation. The reported speedups are up to 10× for the sample inference processing time.

The design of accelerators for cryptographic computations is another frequently targeted domain for building extensions for RISC-V. In article [16], the authors design in-memory-computing (IMC)-adapted instructions for a hash algorithm and measure over 70% improvements in both performance and energy saving with a limited area overhead. Paper [17] presents the design of an RISC-V-based crypto processor for storing sensitive information and performing intermediate cryptography operations which performs 25× better than the software counterpart. The authors of paper [18] add cryptography-accelerating instructions to the RISC-V base ISA for lightweight 64-bit block ciphers. The reported gain in performance can reach 100× and is evaluated using an FPGA prototype. In paper [19], design a secure RISC-V processor capable of always-on encryption to obfuscate the code and pointers. With a 2% area overhead, the designed processor was deployed on Amazon AWS F1 instances and successfully protected a database from attacks. In paper [20], the authors propose a hardware implemented security extension to RISC-V, named FIXER, that provides a defense mechanism against buffer overflow and return-oriented programming (ROP) attacks by using fine-grained control-flow integrity (CFI) checking hardware units. Compared to the existing software-based solutions, FIXER reduces the energy overhead by 60% at a minimal execution time (1.5%) and area (2.9%) overheads. Post-quantum cryptography is approached in article [21], where a new instruction set extension for RISC-V ISA is designed to speed up the computations for CRYSTALS–Kyber and Dilithium algorithms. The results show a modest increase in circuit area (3%) but with a significant speedup of the Kyber and Dilithium functions (20% to 65%) and an energy saving of 20% to 45% compared to software implementation.

The arithmetic processing used in digital signal processing and SIMD processing is often the subject of acceleration. In paper [22], the authors design a vector processing extension for RISC-V which improved the performance by 5.8×compared to the scalar processing and had a 2.6× area overhead. An industrial-grade implementation is presented in paper [23]. A 64-bit high-performance embedded RISC-V processor with SIMD extensions is designed, and its performance is comparable (i.e., within 20% on most benchmarks) with ARM Cortex-A73. In article [24] the authors propose an ISA extension for DSP (Digital Signal Processors) processing in IoT devices with power consumption constraints. The reported speedups are over 50% for the FFT algorithm with an area overhead of 28% compared to the RI5CY implementation of RISC-V. Bit manipulation instructions are added to the RISC-V base instruction set, as indicated in [25]. The FPGA evaluation results show improvements of 4× for Morton encoding/decoding, with a 3× area overhead for the modified ALU.

The customization of communication interface operation is not commonly addressed by systems designers. There is some effort invested in the development of RSIC-V extensions targeted at digital communications, as presented in paper [26]. The authors develop specific instructions that lead to a reduction in clock cycles (needed for certain algorithms involved in 5G communications) of up to 47%, with an area overhead of 25%. Our approach targets the CAN-FD interface operation handling at the instruction set architecture level.

## 3. Architecture of the Proposed Solution

In this chapter, the architecture of the RiscDiag CanFd is presented and explained. In the subsections, details on each important module’s structure and function is provided together with details about the implemented logic.

### 3.1. Architectural Overview

The Real-Time RISC-V-based CAN-FD bus diagnosis tool designed as an SoC contains the following components:

- The *RISC-V core* named *RisCanFd* [6] containing the *CAN-FD Communication Unit/Extension* with a dedicated *ISA extension*;
- The *CAN-FD Bus Diagnosis* module composed of:
  ○ The *CAN-to-UDP Bridge* module (for prebuffering content of the CAN-FD frames, which will be transferred by the *Network Stack* module to the Ethernet PHY and further to the PC);
  ○ The *Network Stack* module (contains an IP with a hardware-implemented communication stack for all necessary protocols);
- The Program and Data Memories of the RISC-V Core;
- The *UART Debugger* module used for controlling the reset of the modules and for write/read operations over the memories;
- *GPO* module (general purpose outputs) for debugging and performance measurements.

**Figure 1:**
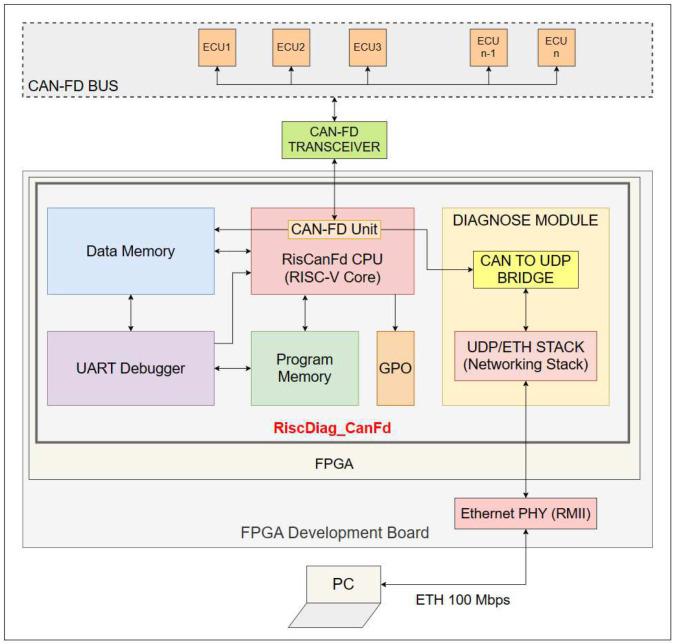
The Architecture of the RISC-V based CAN-FD Bus Diagnosis Tool.

Figure 1 illustrates the architecture of the RiscDiag CanFd SoC in the context of the hardware environment used for deploying and testing the solution. The FPGA loaded with a bit file (i.e., configuration) of the design is Xilinx Artix-7 XC7A100T-1CSG324C, and it is the core component of the Digilent Nexys A7 FPGA Board. The CAN-FD transceiver used for adapting CAN signals to 3V3 TTL signals is Microchip ATA6563, externally connected to the board. The Ethernet physical layer transceiver used is SMSC LAN8720A; it offers an RMII interface, and it is also a component of the FPGA development board.

### 3.2. Architecture of the Hardware Modules

#### 3.2.1. Architecture of the RisCanFd CPU

**Figure 2:**
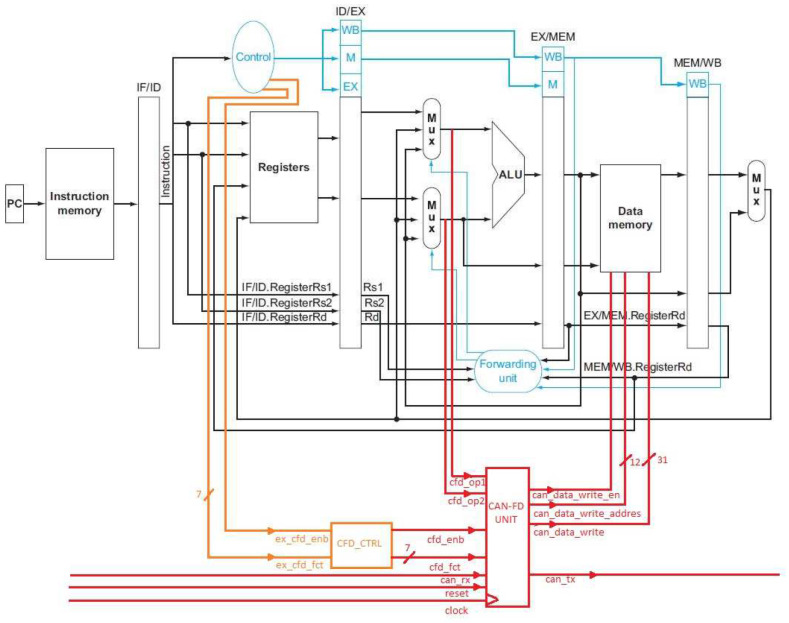
RisCanFd CPU Block Diagram illustrating the CAN-FD Extension [6].

The RisCanFd CPU (Figure 2) was introduced in [6] as an extended RISC-V core. The CPU implements a part of the RV32I ISA variant providing classical instructions such as ADD, SUB, AND, OR, ADDI, LW, SW and BEQ. The core represents a modified version of the Hennessy and Patterson design with a five-stage pipeline, a forwarding unit and no hazard detection unit in the implementation provided as an open-source project [27].

The contributions added to this RISC-V core implementation are the CAN-FD Communication Unit/Extension and the ISA Extension used for configuring and controlling it. The CAN-FD Communication Unit is placed alongside the ALU (Arithmetic-Logic Unit) in the Execution Stage of the core.

##### Architecture of the CAN-FD Communication Unit/Extension

The CAN-FD Communication Unit is the extension of the RISC-V core and represents the module which interacts with the CAN-FD bus for sending and receiving frames. It is controlled by a dedicated ISA Extension set but contains an FSM (Finite State Machine) which allows it to perform tasks independently from other components of the CPU (e.g., transferring the contents of received CAN-FD frames to the Data Memory and to the CAN-to-UDP Bridge).

The core component of the CAN-FD Communication Unit is a CAN-FD engine developed at the Czech Technical University as an “IP core written in VHDL with no vendor-specific libraries needed” [28] and distributed with an open-source license (the only open-source CAN-FD engine available), named CTU-CAN-FD.

The FSM of the CAN-FD Unit controls the CAN-FD engine, described in the paragraph above, by executing the instructions from the RisCanFd ISA Extensions, listed in Table 1, and by independently checking the state of the CAN-FD Engine and performing specific actions:

1. Waiting for the CAN-FD Engine to enter the bus integration state after it was reset and acknowledging the state by writing a flag in the Data Memory;
2. Polling the CAN-FD engine for a received CAN-FD frame writing its contents to the Data Memory and transferring them in parallel to the CAN-to-UDP Bridge for prebuffering before it is transmitted on Ethernet to the PC;
3. Acknowledging that a new CAN-FD frame was received and transferred to the Data Memory and to the CAN-to-UDP Bridge by writing a flag to a specific location;
4. Polling the CAN-FD Engine for any CAN-FD bus error detected and transferring the error-specific information to the CAN-to-UDP Bridge to be sent via Ethernet to the PC.

The CAN-FD Communication Unit writes data directly to the Data Memory and to the CAN-to-UDP Bridge for accelerating the tasks performed by the rest of the RisCanFd CPU with the data received from the CAN-FD bus by classical instructions execution and for accelerating the process of sending all received CAN-FD frames and bus error-associated information to a PC.

Table 1 illustrates the proposed ISA Extension Set designed for controlling the CAN-FD Communication Unit. These instructions use a pseudo-R format, and they have the same opcode but different funct7 field values.

The rd register used as a destination register is not used for the CAN-FD Unit ISA Extension Set; additionally, the ‘xxxxx’ is the notation for an operand register that is not used by the specified instructions. The instructions are using the same opcode but have different funct7 codes for simplifying the decoding logic.

The instruction “cfd_swrst” resets, in a software manner, the CAN-FD Engine inside the unit. The Nominal and Data Bit Rates, which are the most important parameters of the CAN-FD bus, are set using the “cfd_sndbr” instruction. Configuring the mode of operation and enabling the CAN-FD Engine are performed by executing the “cfd_smden” instruction. Setting the ID and other format details of a CAN-FD frame intended to be sent is carried out by executing “cfd_sfmid”. The instruction “cfd_ststp” sets the cycle time of the CAN-FD to be sent if the CAN-FD Engine must work in cyclic timestamped mode. For configuring the payload bytes in the frame to be sent, the instruction “cfd_sbyts” is used, and it offers the possibility of setting four bytes at once (the CAN-FD frame can have a maximum of 64 bytes in payload). For finally sending the CAN-FD frame after the parameters mentioned above are configured, the user must execute “cfd_ssend” [6].

Enabling the reception of CAN-FD frames by repeatedly polling the CAN-FD Engine is carried out by issuing the “cfd_enbrx” instruction. After a CAN-FD frame arrival, the process of transferring it to the Data Memory and to the CAN-to-UDP Bridge will begin immediately [6].

Disabling the CAN-FD Engine is performed by executing the “cfd_disbl” instruction, the latest in the CAN-FD Communication Unit ISA Extension set.

#### 3.2.2. Architecture of the Diagnose Module

The Diagnose module is composed of two modules and has the purpose of sending the received CAN-FD frames and the CAN-FD bus error information to the PC via Ethernet at a bit rate of 100 Mbps. It is composed of the *CAN-to-UDP Bridge* module, which is responsible for sending the data from the CAN-FD Communication Unit to the UDP/ETH Stack and the *UDP/ETH Stack (the Networking Stack)*, an instance of the FC1001_RMII, an IP core implementing the networking stack (DHCP, UDP, IPv4, ARP, ICMP and Ethernet) and exposing an MAC/RMII interface to the Ethernet transceiver used and an UDP server via an AXI stream to the CAN-to-UDP Bridge module. The FC1001_RMII IP core is provided as a black-box netlist IP by FPGA-Cores from Linkoping, Sweden [29]. This module sends the data to the Ethernet Transceiver, which will forward them to the PC.

##### Architecture of the CAN-to-UDP Bridge

**Figure 3:**
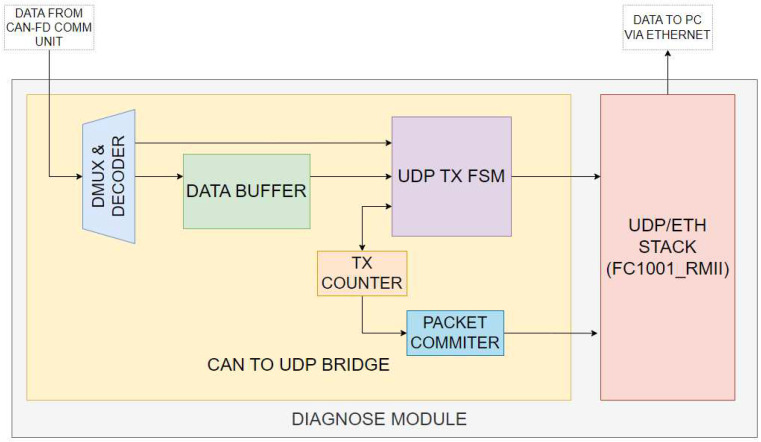
CAN-FD Bus Diagnose Module Architecture.

This module (Figure 3) receives the bytes from the CAN-FD Communication Unit and stores them in the buffer until all of them are received, and, when a complete packet is formed, it sends it to the UDP server inside the UDP/ETH stack for being forwarded to the Ethernet transceiver.

The CAN-to-UDP Bridge contains the *data buffer* (the UDP payload which will be sent on Ethernet), the *demultiplexer* of the data buffer (used for placing the bytes from the CAN-FD Communication Unit), the *UDP transmission FSM* and the *UDP transmission counter*.

For a captured CAN-FD frame, the UDP packet contains the fields of the frame—the arbitration field composed of the ID of the frame, the RTR (Request for Transmission), IDE (Extended ID), FDF (FD Frame) and DLC (Data Length Content) fields and the Data payload—and the timestamp of the frame (on 64 bits). For a bus error situation, the UDP packet contains the error type (bit, CRC, framing, ACK or stuffing) and the error subtype (SOF Bit, arbitration fields, control fields, data, CRC field, ACK bit, EOF bit and others).

The UDP *packet commit module* informs the UDP server that all data have been transmitted, so it can forward them to the UDP client on the PC via Ethernet.

#### 3.2.3. Architecture of the Program Memory

The Program Memory consists of 4096 locations of 32-bit data words and contains the RISC-V machine code, which is executed by the RisCanFd CPU. Memory Read operations are performed asynchronously, and Memory Write operations are performed synchronously. Two entities write into the Program Memory: the *UART Debugger*, with a higher priority, for flashing the SoC with a new firmware, and the *RisCanFd CPU*, with a lower priority. Writing into the Program Memory by the RisCanFd CPU will be used in a future update for adding the CAN-FD bootloader capabilities, which are not available now.

The entities mentioned above are writing/reading the memory using different ports for time efficiency reasons.

#### 3.2.4. Architecture of the Data Memory

The Data Memory consists of 4096 locations of 32-bit data words. Memory Read operations are performed asynchronously, and Memory Write operations are performed synchronously. Three entities write into the Data Memory with the following priorities (where a lower number represents a higher priority):

1. *UART Debugger* for directly writing sets of data, which is used in application-specific scenarios (e.g., configurations for the CAN-FD Communication Unit, format and contents of the CAN-FD frame intended to be sent later, masks and patterns for computations with CAN-FD-received frames data, the IP address that is used by the UDP server inside the diagnose module, etc.);
2. *CAN-FD Communication Unit* for efficiently bringing to the memory the format and content of every captured CAN-FD frame or other flag values. These values are written without the intervention of the RisCanFd CPU in an emulated DMA manner;
3. RisCanFd CPU for storing computations results [6];
4. The entities mentioned above are writing/reading the memory using different ports for time efficiency reasons.

#### 3.2.5. Architecture of the UART Debugger

The UART Debugger is a module that interacts with the RisCanFd CPU, with the Program Memory and with the Data Memory [6]. This module resets all SoC submodules, writes/reads to/from the Program Memory (flashing and verifying a new firmware), writes the Data Memory with values used by the program flashed and reads it for obtaining execution results and for debugging purposes.

**Figure 4:**
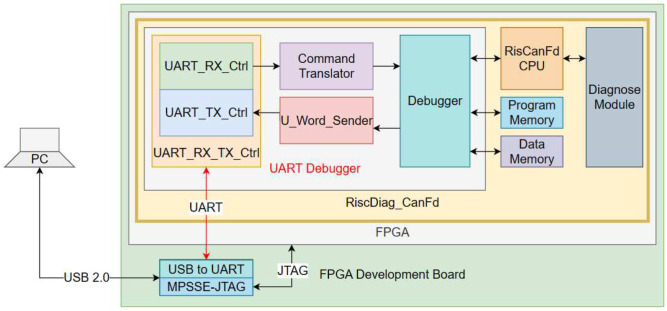
The Expanded Architecture of the UART Debugger.

The UART Debugger (Figure 4) contains the *UART RX-TX engine*, the *Command Translator*, the *U-Word-Sender (Unsigned 32-bit word)* and the *Debugger*. The UART RX submodule forwards the command (on eight bytes) to the Command Translator which controls the Debugger. The Debugger sends the requested data or a success/failure code to the U-Word-Sender, which transforms this information into the four-bytes response and forwards it to the UART TX submodule [6].

The functionalities of the UART Debugger are accessed by the user within the RisCanFd IDE, composed of a dedicated assembler which supports the ISA Extension Set and a GUI Application [6].

The UART RX-TX Control module uses a hardcoded baud rate of 921,600 bps, one of the highest reliable baud rates for UART communication (Universal Asynchronous Receiver–Transmitter). The UART protocol was chosen because the FPGA development board contains an FTDI 2232H, “a USB 2.0 Hi-Speed (480 Mb/s) to UART/FIFO IC” [30] with two communication pipes, one using the MPSSE (Multi-Purpose Synchronous Serial Engine) for flashing the bitfile of the FPGA via JTAG and other a spare UART FIFO, which can be used by the designer using the development board. Because writing/reading into/from Program and Data Memory are not time-critical operations for the end user, this spare UART FIFO channel was used for controlling the UART Debugger module of the RiscDiag CanFd.

### 3.3. Description of the Firmware used by the SoC for CAN Bus Diagnosis

The firmware used by the RiscDiag_CanFd for achieving the goal of capturing all the CAN-FD frames present on the bus and all the bus errors and sending the information on UDP via Ethernet to the PC follows the following steps:

1. Issuing software reset to the CAN-FD Communication Unit;
2. Configuring the Nominal and Data Bit Rates of the CAN-FD Engine to the values of the bus to be diagnosed;
3. Setting the mode of operation of the CAN-FD Engine (ACK of the received frames disabled and bus monitoring mode enabled—passive CAN-FD node on the bus) and enabling the CAN-FD Engine;
4. Waiting in a loop and checking a specific Data Memory location flag value for validating that the CAN-FD Engine inside of it entered the bus integration state [28];
5. Issuing to the CAN-FD Communication Unit the command to start listening for CAN-FD frames (this operation also ensures the detection of the bus error situations);
6. Checking if any CAN-FD frame is captured by permanently polling the value of a specific Data Memory location written by the CAN-FD Unit when receiving frames without the intervention of the RisCanFd CPU;
7. If a CAN-FD frame is captured, the instruction for starting to listen again for frames is issued to the CAN-FD Communication Unit; the RisCanFd CPU does not perform supplementary actions for sending the frames to the diagnose module for sending them on Ethernet because at a CAN-FD frame receival or at a bus error detection, all the information is sent directly by the CAN-FD Communication Unit to the CAN-to-UDP Bridge inside the diagnose module, which functions independently and forms the UDP packet to be sent to the PC.

### 3.4. Implementation of the Hardware Solution

The RiscDiag CanFd SoC was designed in using the VHDL (VHDL-2008) and Verilog (Verilog-2001) hardware description languages under Vivado 2022.1 IDE. Most of the modules were written in VHDL—the RISC-V core and its submodules (the exception is the CAN-FD Unit), the Program and Data Memories, etc., and a few were written in Verilog—the CAN-FD Communication Unit/Extension, the UART Debugger and its submodules, the CAN-to-UDP Bridge, etc.

For validating all the instructions in Extended ISA of the CAN-FD Unit and its integration into the RISC-V core, simulations were performed using the Vivado Simulator targeting mixed language (VHDL + Verilog) with simulation files written in Verilog.

### 3.5. Architecture of PC Software Application

For showing and saving into trace files the captured CAN-FD frames and the bus errors information, a PC GUI application was necessary. The GUI Application, named *RiscDiag_CanFd_App*, was developed in C# under .NET Framework 4.8. It uses a DLL (Dynamic Linked Library) called *RiscDiag_CanFd_Library* containing the *UDP Driver* named *RiscDiag_CanFd_Driver*.

#### Architecture of the RiscDiag_CanFd Library

The library contains classes for the UDP Packets, for the CAN-FD decoded frames, for the CAN-FD frames cache and for the main application logic. *UdpFrame* is a serializable class used for storing the UDP frames received from the UDP server inside the diagnose module of the SoC. The *CanFrame* class is designed for decoding the information inside the UdpFrame and storing them into specific CAN-FD frame fields. This class also has methods for exporting the CAN-FD frames information into string or TreeNode objects for listing the frames contents on the GUI application. The *CanFrameCache* class stores the CAN-FD frames and monitors their number; when there are 500 frames stored, a thread for saving them into TXT or XML files is instantiated and started.

The core class of the library is the *RiscDiag_CanFd Driver*. The driver is responsible for starting a UDP socket as a UDP client using the IP address and port of the UDP server in the SoC, receiving the UDP packets from the SoC, converting the information from the UDP packet into CAN-FD-specific fields, using this information to form a TreeNode to be added on the TreeView of the GUI Application and saving the CAN-FD frames and Bus Error information in TXT or XML files.

**Figure 5:**
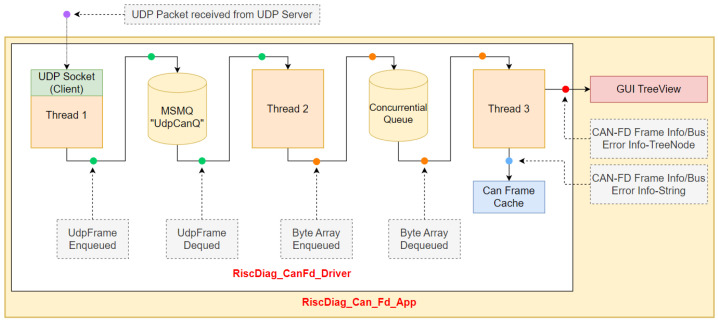
Information Flow in the Main Application of the Driver.

The main application of the driver consists of three threads which are described in this section. Even though, initially, there were just two threads, one for receiving UDP packets with the socket and enqueueing them into a ConcurrentialQueue and one for dequeuing them, converting them to CAN-FD frames and showing them on the GUI, this approach created a loss of UDP packets on the way from the Socket to the ConcurrentialQueue. Because of this problem, another MSMQ (Microsoft Message Queue) was inserted into the logic together with another thread. The threads used and illustrated in Figure 5 by the driver have the following roles:

1. The first thread represents the UDP client which reads the UDP packets with a socket opened at the SoC endpoint using its connection data (IPv4 address and port) and enqueues them into the MSMQ (Microsoft Message Queue), called “UdpCanQ”;
2. The second thread dequeues the UDP packets and enqueues their payload represented as byte array objects to an .NET Framework ConcurrentialQueue;
3. The third thread dequeues the byte array objects from the ConcurrentialQueue and extracts the CAN-FD frame or CAN-FD bus error-associated information for building the TreeNode, which are displayed in the GUI and which are stored in the CanFrameCache for writing them into XML or TXT files.

Because the ConcurrentialQueue provided by the .NET Framework caused a loss of UdpFrame instances, the MSMQ was introduced for “slowing down” the information flow using a reliable queueing instrument offered by the Windows operating system.

## 4. Performance Evaluation

### 4.1. Evaluation Criteria for the Bus Diagnosis Tool

In this subsection, the criteria used for evaluating the CAN-FD bus diagnosis tool are discussed. The point is not necessarily to prove that the proposed solution performs better than other solutions available on the market but to demonstrate that it will properly and correctly work in all possible use cases and scenarios. These criteria are:

1. The number of CAN-FD frames lost for different bus loads, which *must be 0* for a busload of 100%; the bus load represents the percentage of time during which the bus is populated with messages or, in general, with useful data—for example, if in 1 ms there is a sequence of five cyclical messages during a 150 µs transmission for each of them and every message is followed by a break of 50 µs, the bus load is calculated with the formula presented in Equation (1):

(1) $$bus_{load}=\frac{\Delta t_{bus_{occupied}}}{\Delta t_{bus_{occupied}}+\Delta t_{bus_{free}}}=\frac{5\times 150\;µs}{\left(5\times 150\;µs\right)+\left(5\times 50\;µs\right)}=\frac{150\;µs}{200\;µs}=0.75=75\%\;$$

2. The timestamp precision for the valid captured CAN-FD frames; the trace containing all CAN-FD frames information packets must contain their timestamp for being able to diagnose every problem, which occurred in the Distributed Embedded System, which uses CAN-FD as the data exchange environment; the difference between a cycle time of CAN-FD frames measured with the diagnose tool and a cycle time measured with an oscilloscope/logic analyzer must be as small as possible.
3. The time duration needed by the diagnosis tool for sending the captured CAN-FD frames and the bus error situations to the PC, as well as the delay between the moment the information of the captured frame is sent to the PC and the moment another frame arrives on the bus and has to be captured; it is required that the *diagnosis tool* finishes sending the captured information *before another frame has been completely transmitted* on the bus; in an ideal scenario, the diagnosis tool may send all the captured information within a CAN-FD frame *even before the next CAN-FD frame starts to be transmitted on the bus*; for evaluating in the perspective of this criteria, the time needed for sending the captured information from the bus on the Ethernet and the time passed between all data had been transmitted, and the diagnose tool is *waiting* before another CAN-FD frame arrives on the bus.

### 4.2. Test and Measurement Setup

For testing the RISC-V-based CAN-FD diagnosis tool, a bus with several CAN-FD-capable devices and an oscilloscope or at least a logic analyzer capable of decoding the CAN-FD frames is required.

**Figure 6:**
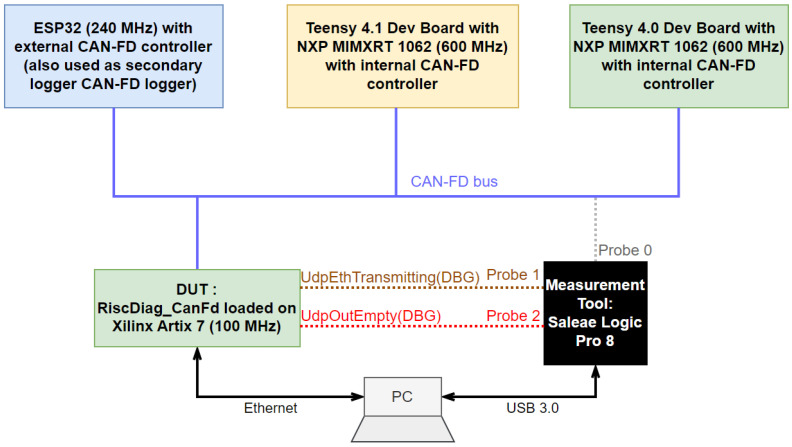
The Test Setup.

The test setup, illustrated in Figure 6, contains four devices on the CAN-FD bus and a measurement tool. The devices on the bus are two NXP MIMXRT 1062 ARM Cortex-M7 microcontrollers (with internal CAN-FD controllers as memory-mapped peripherals), one ESP32 microcontroller (with an external CAN-FD controller, also used as a secondary CAN-FD logger) and the DUT (Device Under Test); the FPGA is loaded with the RiscDiag CanFd hardware configuration. The DUT is passive on the CAN-FD bus—it does not acknowledge frames and it does not send frames.

The tool used for performing tests and measurements is the Saleae Logic Pro 8 logic analyzer and protocol decoder.

The two ARM Cortex-M7 NXP MIMXRT 1062s running at 600 MHz have an internal classical CAN-FD memory-mapped peripheral, and their purpose in the setup is to simulate the exchange of messages at a 100% busload, a scenario imitating the communication of ECUs in a vehicle in the automotive industry. The ESP32 running at 240 MHz is used for comparing, in terms of time, the efficiency of the use of external and internal CAN-FD controllers and also has the purpose of the secondary CAN-FD frames tracer on the bus.

Because of the high prices of the protocol decoding licenses for oscilloscopes, a high-end logic analyzer with protocol decoder capacities was chosen as the measurement and decoding tool. For about EUR 1000, it offers digital acquisition for signals with a maximum frequency of 100 MHz and analog acquisition for signals with a maximum frequency of 5 MHz. The PC Software provides a reasonable number of protocol decoder extensions. The protocol decoder for CAN-FD was not provided by the producer, so an open-source CAN-FD extension was developed and published as an open-source project [31].

### 4.3. Evaluating the Lost Frames Rate of the Solution

#### 4.3.1. Test Conditions and Methodology

For validating that the CAN-FD diagnosis tool does not lose any frame, two scenarios were elaborated upon:

- Sending from one Teensy 4.1 MCU 65,536 CAN-FD frames on the bus on a busload of 100% (no breaks between frames);
- An exchange of 65,536 CAN-FD frames between Teensy 4.1 and Teensy 4.0 MCUs: for an agreed frame, Teensy 4.1 increments a fixed position counter placed on two bytes (for 65,536 frames exchanged) if the counter value received from Teensy 4.0 is odd, and Teensy 4.0 increments the 16-bit counter only if the counter value received from Teensy 4.1 is even; this approach simulates a request–reply communication scenario.

For the tests, all CAN-FD frame lengths were used (0, 1, 2, 3, 4, 5, 6, 7, 8, 12, 16, 20, 24, 32, 48 and 64). The scenarios were tested for two combinations of Nominal and Data Bit Rates—*500 kbps*–*2 Mbps*, the most used combination in the Automotive Industry, and *1 Mbps*–*4 Mbps*, the highest values supported by the Teensy Devices’ internal CAN-FD controllers. The Data Bit Rates of 5 and 8 Mbps are not largely adopted and not supported by all CAN-FD Transceivers; 1 Mbps is the maximum value for the Nominal Bit Rate of CAN-FD Communication (the same as the Arbitration Rate).

Both test scenarios were run 50 times. All the traces generated by the RiscDiag_CanFd_App were parsed, and the vehiculated counter values were verified for checking if any frame was lost/not captured by the RiscDiag_CanFd solution and determining the lost frame rate.

#### 4.3.2. Test Results and Discussion

The test results listed in Table 2 prove that, for the combinations of Nominal-Data Bit Rates used and for all possible CAN-FD frame lengths, the RISC-V-based CAN-FD bus diagnosis tool designed does not lose any frame, so the first evaluation criterion described in Section 4.1 is satisfied.

### 4.4. Evaluating the Timestamp Precision of the Solution

#### 4.4.1. Context for Timestamp and Cycle Times

CAN and CAN-FD have a collision avoidance mechanism based on the priorities derived from frames’ IDs (a lower ID for a higher priority), and it can easily be used in an event-triggered paradigm for Embedded Computing Applications with tens of MCUs on the bus. If a node/MCU loses arbitration because the frame it tries to send on the bus has a higher ID than another “winning” node, it tries a retransmission for a configurable number of times.

For preventing frame losses due to nodes waiting to win arbitration on the bus forever and other timing hazards such as OS tasks not having their input data at a specified moment, Automotive Real-Time Embedded Applications with buses containing tens of nodes implement methods for shifting from an event-triggered paradigm to a time-triggered paradigm. Both paradigms are thoroughly described in the book [32].

For time-triggered events and for each OS task of each MCU on the bus to know exactly when it will have to send/receive frames on/from the bus, each MCU saves “databases” of frames information in its memory, one of these being the cycle time for sending/receiving frames. Other methods such as Time-Triggered CAN (TTCAN) [33,34] include more elaborated architectures and mechanisms for achieving the real-time behavior of the CAN/CAN-FD Distributed Embedded Systems.

Taking the oscillograms of the Automotive CAN/CAN-FD bus reveals a 100% bus load because there are hundreds or thousands of frames arrived upon at a precise time known by each node “interested” in sending or receiving that frame as the cycle time. The resolution of the cycle time is the millisecond. Each CAN/CAN-FD frame has a cycle time of 1 ms, 10 ms, etc.

#### 4.4.2. Test Conditions and Methodology

For evaluating the precision of the timestamps provided by the proposed solution for the captured frames, the cycle times of the frames with the same IDs were measured by computing the differences between the timestamps of the sniffed frames and comparing the results with the cycle times measured by the logic analyzer.

The tests were performed at the maximum bit rates supported by all the nodes on the bus, a Nominal Bit Rate of 1 Mbps and a Data Bit Rate of 4 Mbps. Nine groups of measurements were performed for CAN-FD frames with a maximum length of 64 bytes (DLC field with the value 15). The programmed cycle times of the frames go from 10 ms to 90 ms, with a step of 10 ms.

For each measurement group, 100 differences between the timestamps were computed after parsing the trace files saved by the PC Application of the solution. The results were compared with the cycle times measured with the logic analyzer using its automation API.

#### 4.4.3. Test Results Discussion

Each cycle time measurement from Table 3. represents the average of 100 samples of cycle times measured for each theoretical cycle time in the first column of the table. Considering that the absolute error values are in the same order of magnitude and they are under 0.001%, it may be said that the solution can be used for offering a precise image about the history of events on the CAN-FD bus. The increasing difference between the two sources of measurements obtained from increasing the cycle time comes from the fact that the solution does not implement any mechanism of clock synchronization or clock drift correction. This solution will be included in a further implementation.

### 4.5. Evaluating the Time Effort and Efficiency for the Communication over Ethernet to a PC

In this subsection, the proposed solution is evaluated in terms of the time effort needed for sending the captured CAN-FD frames and CAN-FD bus error information to a PC via Ethernet using the UDP transport layer protocol. There are two metrics that are used:

- The time effort for forming the Ethernet package containing the 82 bytes of useful data and sending it over Ethernet to the PC;
- The time duration between the Ethernet transmission is finished, and another CAN-FD frame arrives on the bus (the resting or “recovery” time after one captured CAN-FD frame is sent to the PC and another one starts to be out of the bus).

#### 4.5.1. Test Conditions and Methodology

The time effort for packing the useful data into an Ethernet frame is measured with the Logic Analyzer based on the debug signal UdpOutIsEmpty illustrated in Figure 6. This duration is equal to the time during which this signal has the logic value of ‘0′.

The amount of time needed for the Ethernet packet to be transmitted from the Ethernet transceiver to the PC can be calculated by considering the dimensions of the packets, which encapsulate the useful data. The useful data sent by the solution comprise 82 bytes, and the UDP protocol has a header of 8 bytes [35] (Equation (2)). The result is that the IPv4 packet has 90 bytes of payload plus the header consisting of 20 bytes [36] (Equation (3)). The Ethernet Packet has a data payload of 110 bytes and a header of 18 bytes [37], for a total of 128 bytes (Equation (4)) transmitted with a speed of 100 Mbps, resulting in an Ethernet transmission time of 10.24 µs (Equation (5)).

(2) $$Bytes_{UDP}=Bytes_{UDP_{Header}}+Bytes_{UDP_{Payload}}=8\;\mathrm{bytes}+82\;\mathrm{bytes}=90\;\mathrm{bytes}$$

(3) $$Bytes_{IPv4}=Bytes_{IPv4_{Header}}+Bytes_{IPv4_{Payload}}=Bytes_{IPv4_{Header}}+Bytes_{UDP}=20\;\mathrm{bytes}+90\;\mathrm{bytes}=110\;\mathrm{bytes}$$

(4) $$Bytes_{ETH}=Bytes_{ETH_{Header}}+Bytes_{ETH_{Payload}}=Bytes_{ETH_{Header}}+Bytes_{IPv4}=18\;\mathrm{bytes}+110\;\mathrm{bytes}=128\;\mathrm{bytes}$$

(5) $$\Delta_{t_{ETH_{transmission}}}=\frac{Bytes_{ETH}\times \frac{8\;\mathrm{bits}}{\mathrm{byte}}}{\frac{100\;\mathrm{Mbits}}{s}}=\frac{128\;\mathrm{bytes}\times \frac{8\;\mathrm{bits}}{\mathrm{byte}}\;}{\frac{10^{8}\;\mathrm{bits}}{s}}=\frac{1024\;\mathrm{bits}}{\frac{10^{8}\;\mathrm{bits}}{s}}=10.24\;\mu s$$

The recovery time between an Ethernet transmission and the next CAN-FD frame that must be captured is measured as the delay between UdpOutIsEmpty, which changes its value from logical ‘0’ to logical ‘1’, and the SOF bit of the next CAN-FD frame.

The tests and measurements are performed using the two combinations of bit rates used and explained previously: 500 kbps–2 Mbps and 1000 kbps–4 Mbps at a 100% busload (no breaks between CAN-FD frames). CAN-FD frames with all possible lengths were used for validating that the length of CAN-FD frames does not impact either the time effort for transferring the data to the Ethernet Transceiver or the recovery time.

#### 4.5.2. Test Results Discussion

The results of the measurements performed for determining the time effort needed by the solution for embedding the captured CAN-FD frame data or bus error information into an Ethernet packet are listed in Table 4.

The measurements confirm that the effort time described above is not impacted by the CAN-FD communication bit rates nor the CAN-FD frame length. This result is the consequence of using the same UDP payload length for all the captured CAN-FD frames for ensuring a deterministic behavior. Additionally, the jitter values prove a deterministic behavior.

**Figure 7:**
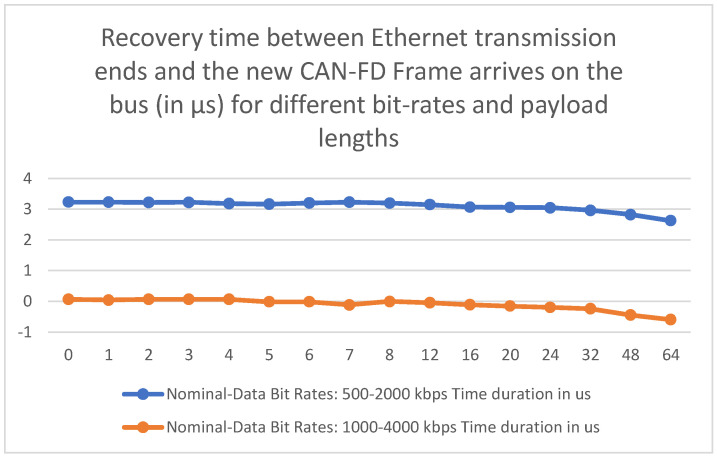
Plot of recovery time measurement results from Table 5.

Another time measurement necessary for validating once more that the solution is fast enough to send the information contained in every captured CAN-FD frame is the recovery time between the termination of a UDP packet transmission and the SOF bit of the next CAN-FD frame to be captured. The results for these measurements are listed in Table 5 and plotted in the chart displayed in Figure 7.

For using Nominal-Data Bit Rates combination 500 kbps–2 Mbps (most used in the Automotive Industry), it can be observed that the transmission of all the data associated with a captured CAN-FD frame is finished within 2.624–3.232 µs before the next CAN-FD frame to be captured starts to be sent on the CAN-FD bus. Taking this into account, it may be said that the solution meets the ideal criterion described in the third point from the beginning of the chapter.

For the case of using the 1–4 Mbps values of the Nominal-Data Bit Rates negative delay values, it can be observed that for capturing a CAN-FD frame with a payload of at least five bytes, all of the frame’s information will be transmitted to the Ethernet PHY in less than 1 µs after the SOF bit of the next CAN-FD frame starts to be transmitted. Even in this case, it is concluded that the solution will not drop any CAN-FD frame Ethernet transmission, because a new Ethernet transfer will start after the whole CAN-FD frame becomes present and acknowledged on the bus. Considering that the SOF bit is transmitted at a Nominal Bit Rate of 1 Mbps, the SOF bit of the next CAN-FD is not even completely sent on the bus (596 ns out of 1 µs) when the proposed solution finishes sending the current frame information to the Ethernet PHY.

In both cases, the value of delay between ending an Ethernet transmission and the arrival of a new CAN-FD frame decreases with the increase in the CAN-FD frame payload length because the CAN-FD Unit has more data to transmit to the CAN-to-UDP Bridge inside the Diagnose Module.

The interpretation of these measurement results confirms that the time effort of the proposed solution is low enough for sending the captured data to the PC without dropping any captured CAN-FD frame.

The CAN-FD frame acquisition cycle starts when the ACK bit of the frame appears on the bus and ends when all its information is sent on the Ethernet lines. The acquisition cycle time can be considered the CAN-FD—Ethernet transfer delay. The measurements for the acquisition cycle were performed for both Nominal-Data Bitrate combinations mentioned above, with a CAN-FD payload of all lengths and a busload of 100%. The results of the CAN-FD frame acquisition cycle times are listed in Table 6. Comparing the duration of the acquisition cycle with its jitter values proves the real-time behavior of the solution.

**Figure 8:**
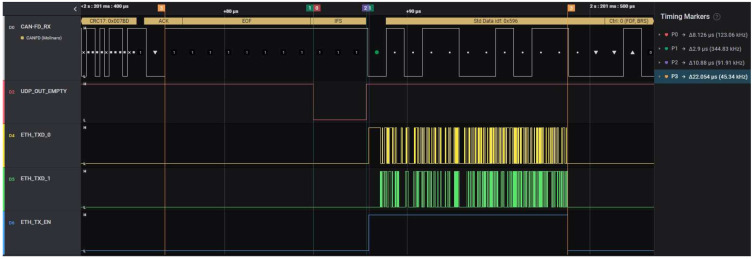
Oscillogram of the CAN-FD frame acquisition cycle captured with the logic analyzer.

The main difficulty in measuring the acquisition cycle time was capturing the moment when the Ethernet transmission ends. Because of the lack of an Ethernet decoding license for the oscilloscope and because the logic analyzer is not capable of differential measurements and Ethernet decoding, the RMII signals between the FPGA and the Ethernet transceiver were used. The two-bit-wide ETH_TXD parallel bus used for transferring the Ethernet package to the transceiver and the ETH_TX_EN control signal were used for capturing the moment when the Ethernet transmission ends. These signals, together with the CAN-FD decoded frame, are illustrated in the oscillogram printed in Figure 8. The capture is performed for the worst-case scenario, where the CAN-FD busload is 100%, the data length of the frames’ payload is 0 and the Nominal Bitrate is the highest possible, 1 Mbps; these are all conditions that are necessary for proving not only that the solution is not losing frames but that it has a deterministic and real-time behavior, as presented in Table 6.

The results from Figure 8 also show that the content of the latest captured CAN-FD frames finishes being transferred on Ethernet before the ID of the next CAN-FD frame is completely output on the CAN-FD bus. The time duration marked in the oscillogram with P0 is the time needed for transferring the CAN-FD frame content from the CAN-FD Unit to the CAN-to-UDP Bridge and represents the reaction time of the RisCanFd CPU for CAN-FD frames. The duration marked with P1 represents the time effort for forming the Ethernet frame by the UDP/ETH Stack module; the measurements for this delay were presented in The results of the measurements performed for determining the time effort needed by the solution for embedding the captured CAN-FD frame data or bus error information into an Ethernet packet are listed in Table 4.

The duration marked with P2 represents the time needed for the Ethernet transmission, measured as the duration of the ETH_TX_EN signal (Ethernet transmission enabled) and computed theoretically above in Equations (2)–(5). The duration marked with P3 represents the duration of the full CAN-FD acquisition cycle, starts with the ACK bit of the CAN-FD frame and ends with the negative edge of the ETH_TX_EN signal; the measurements of these delays are listed above in Table 6.

**Figure 9:**
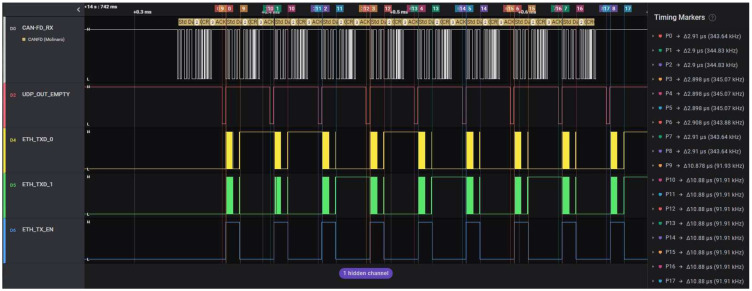
Oscillogram presenting multiple CAN-FD acquisition cycles.

The oscillogram in Figure 9 shows multiple CAN-FD acquisition cycles with duration measurements for packing the data into the Ethernet frames (time delay markers P0 to P8) and for Ethernet transmissions (time delays marked P9 to P17).

### 4.6. Evaluating the Performance of the CAN-FD RISC-V Extension

After validating the proposed solution through tests and measurements, in this subsection, the focus is moved to the performance of the CAN-FD Communication Unit, the RISC-V extension of the RisCanFd CPU.

The evaluation criterion is the time necessary for capturing a CAN-FD frame and performing CPU operations with its content. The results are compared with similar ones from MCUs using integrated memory-mapped CAN-FD controllers or MCUs with an external CAN-FD controller connected to them.

#### 4.6.1. Test Conditions and Methodology

The measurements refer to the time amount needed for three devices (RiscDiag CanFd, Teensy 4.1 NXP MIMXRT 1062 with an internal CAN-FD controller and ESP32 with an external CAN-FD controller) in performing the following task: capturing a CAN-FD frame, comparing its ID and first four payload bytes with a known pattern and triggering a digital transition from ‘0’ to ‘1’ for a data pattern complete match and a transition from ‘1’ to ‘0’ otherwise. The reaction time is measured as the delay between the ACK bit of the frame and the triggered output transition performed after each device finishes analyzing its data.

The test is performed using the Nominal-Data Bit Rates 1–4 Mbps with a CAN-FD length of eight bytes.

#### 4.6.2. Test Results Discussion

**Figure 10:**
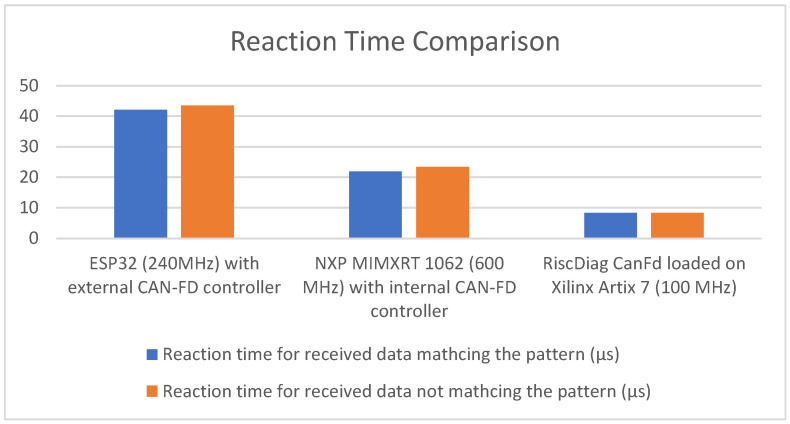
Reaction Time Comparison Chart.

Analyzing the results listed in Table 7 and plotted in the chart from Figure 10 leads to the following conclusions:

- The slowest device is the MCU connected to an external CAN-FD controller because of the communication overhead of the SPI bus used for sending the captured CAN-FD frame to the MCU.
- The almost three-times-better performance achieved by the RiscDiag CanFd even while running at a six-times-lower frequency compared to NXP MIMXRT 1062 proves the superiority of application-specific CPU extensions over classical communication peripherals of microcontrollers.

## 5. Conclusions

The contribution provided by this paper is introducing an SoC based on an RISC-V extended core capable of tracing and diagnosing CAN-FD buses. The CAN-FD extension is a communication unit working independently but controlled by an extended instruction set. The extension works in parallel with ALU for optimizing the reaction time and for capturing CAN-FD frames and bus error information. The proposed SoC is capable of UDP communication via Ethernet for being accessed by the PC of the end user. The software application of the solution displays and saves into files all the information captured from the CAN-FD bus. The performance of the solution includes deterministic behavior, with a jitter of the acquisition cycle under 10 ns, a 0% frame loss rate and a timestamp error under 0.001%; all tests were performed for the highest bit rates and a 100% busload of the CAN-FD communication.

The results and explanations show that the target of designing a CAN-FD bus diagnosis tool with real-time behavior was achieved, and the solution can be used for developing and testing the Distributed Embedded Systems which use the CAN-FD communication bus as the data exchange channel. The design sources for using this project are available with an open-source license on GitHub in a repository called “RiscDiagCanFd”.

Another important aspect emphasized by the tests and measurements presented is that an RISC-V CPU with an application-specific extension can be more efficient for time-critical applications than classical microcontrollers with memory-mapped peripherals. The application-specific hardware and application-specific parallelization represent a solution for the physical and technological limitations that hardware designers have confronted in the last two decades, and such architectures targeting specific applications such as IoT, Neural Networks or Ultra-High-Speed Communication may be an alternative to classical general-purpose designs.

## Figures and Tables

**Table 1:** The ISA Extension Set used for Controlling the CAN-FD Communication Unit [6].

instr_name	funct7	rs2	rs1	opcode
cfd_swrst	0000001	xxxxx	xxxxx	1111000
cfd_sndbr	0000010	$_DBR	$_NBR	1111000
cfd_smden	0000011	xxxxx	$_mode	1111000
cfd_sfmid	0000100	$_frame_ID	$_frame_format	1111000
cfd_ststp	0000101	$_cycle_time_L	$_cycle_time_U	1111000
cfd_sbyts	0000110	$_data_word	$_data_offset	1111000
cfd_ssend	0000111	xxxxx	xxxxx	1111000
cfd_enbrx	0001000	xxxxx	xxxxx	1111000
cdf_disbl	1111111	xxxxx	xxxxx	1111000

**Table 2:** Test Results for Measuring the Lost Frame Rate in the various scenarios described above.

DLC Field of the CAN-FD Frame	Length of the CAN-FD Frame Data Payload	Lost Frame Rate (%) at 500–2000 kbps	Lost Frame Rate (%) at 1000–4000 kbps
0	0	0	0
1	1	0	0
2	2	0	0
3	3	0	0
4	4	0	0
5	5	0	0
6	6	0	0
7	7	0	0
8	8	0	0
9	12	0	0
10	16	0	0
11	20	0	0
12	24	0	0
13	32	0	0
14	48	0	0
15	64	0	0

**Table 3:** Test Results for Comparing Cycle Times measured using the Proposed Solution with the results from the Saleae Logic Analyzer.

Database Cycle Time (ms)	Cycle Time Measurements from Saleae (ms)	Cycle Time Measurements from RiscDiag_CanFd (ms)	Difference (ms)	Difference (ns)	Absolute Error (%)
10	9.992663	9.992702	0.000039	39	0.000390286
20	20.156012	20.1561	0.000088	88	0.000436594
30	30.106394	30.1065	0.000106	106	0.000352085
40	40.274748	40.2749	0.000152	152	0.000377408
50	50.149134	50.1493	0.000166	166	0.000331013
60	59.936526	59.9368	0.000274	274	0.00045715
70	70.62186	70.6222	0.000340	340	0.000481437
80	80.677238	80.6776	0.000362	362	0.000448702
90	89.859636	89.86	0.000364	364	0.000405076

**Table 4:** Time effort of the solution for forming the Ethernet frame containing the 82 bytes.

CAN-FD Frame Payload Length	Nominal-Data Bit Rates: 1000–2000 kbps		Nominal-Data Bit Rates: 1000–4000 kbps	
	Time Duration (µs)	Jitter (ns)	Time Duration (µs)	Jitter (ns)
0	2.902706	0.23053	2.902471	0.12046
1	2.902571	0.36498	2.902769	0.17818
2	2.902750	0.18641	2.902556	0.03550
3	2.902375	0.56141	2.902632	0.04053
4	2.903500	0.56359	2.902100	0.49105
5	2.903875	0.93859	2.902588	0.00282
6	2.902625	0.31141	2.902105	0.48579
7	2.902875	0.06141	2.902000	0.59105
8	2.903375	0.43859	2.902667	0.07562
12	2.903000	0.06359	2.902615	0.02433
16	2.903286	0.34930	2.903250	0.65895
20	2.902762	0.17451	2.903111	0.52006
24	2.902250	0.68641	2.901818	0.77287
32	2.903765	0.82829	2.902857	0.26609
48	2.902824	0.11288	2.903222	0.63117
64	2.902444	0.49197	2.902696	0.10460

**Table 5:** Delay between the captured CAN-FD frame information sent on Ethernet and the beginning of a new CAN-FD frame to be captured.

CAN-FD Frame Payload Length	Nominal-Data Bit Rates: 500–2000 kbps (Time Duration in µs)	Nominal-Data Bit Rates: 1000–4000 kbps (Time Duration in µs)
0	3.232	0.066
1	3.222	0.044
2	3.216	0.06
3	3.218	0.066
4	3.18	0.062
5	3.162	−0.014
6	3.2	−0.014
7	3.222	−0.12
8	3.198	−0.002
12	3.146	−0.044
16	3.066	−0.11
20	3.06	−0.154
24	3.042	−0.194
32	2.958	−0.24
48	2.82	−0.446
64	2.624	−0.596

**Table 6:** Results of CAN-FD frame acquisition cycle time measurements.

CAN-FD Frame Payload Length	Nominal-Data Bit Rates: 1000–2000 kbps		Nominal-Data Bit Rates: 1000–4000 kbps	
	Acquisition Cycle (µs)	Jitter (ns)	Acquisition Cycle (µs)	Jitter (ns)
0	22.691706	7.73053	22.703471	4.37954
1	22.699571	0.13502	22.700769	1.67818
2	22.696750	2.68641	22.697556	1.53550
3	22.703375	3.93859	22.699632	0.54053
4	22.694500	4.93641	22.706100	7.00895
5	22.705875	6.43859	22.704588	5.49718
6	22.697625	1.81141	22.698105	0.98579
7	22.702875	3.43859	22.691000	8.09105
8	22.693375	6.06141	22.695667	3.42438
12	22.706000	6.56359	22.702615	3.52433
16	22.701286	1.84930	22.706250	7.15895
20	22.694762	4.67451	22.695111	3.97994
24	22.706250	6.81359	22.695818	3.27287
32	22.699765	0.32829	22.701857	2.76609
48	22.701824	2.38712	22.693222	5.86883
64	22.695444	3.99197	22.693696	5.39540

**Table 7:** Reaction time measurement results.

Device Used	Reaction Time for Received Data Matching the Pattern (µs)	Reaction Time for Received Data Not Matching the Pattern (µs)
ESP32 (240 MHz) with an external CAN-FD controller	42.106	43.519
NXP MIMXRT 1062 (600 MHz) with an internal CAN-FD controller	21.876	23.423
RiscDiag CanFd loaded on Xilinx Artix 7 (100 MHz)	8.284	8.312

## Data Availability

Not applicable.
